# Modulation of hepatic miRNA expression in Atlantic salmon (*Salmo salar*) by family background and dietary fatty acid composition

**DOI:** 10.1111/jfb.14649

**Published:** 2021-01-11

**Authors:** Tone‐Kari K. Østbye, Nardos T. Woldemariam, Camilla E. Lundberg, Gerd M. Berge, Bente Ruyter, Rune Andreassen

**Affiliations:** ^1^ Nofima (Norwegian Institute of Food, Fisheries, and Aquaculture Research) Ås Norway; ^2^ Department of Life Sciences and Health, Faculty of Health Sciences OsloMet – Oslo Metropolitan University Oslo Norway

**Keywords:** Atlantic salmon, diet, lipid metabolism, miRNA, small‐RNA sequencing

## Abstract

This study finds significant differences in hepatic fatty acid composition between four groups of Atlantic salmon (*Salmo salar*) consisting of offspring from families selected for high and low capacities to express the delta 6 desaturase isomer b and fed diets with 10% or 75% fish oil. The results demonstrated that hepatic lipid metabolism was affected by experimental conditions (diet/family). The fatty acid composition in the four groups mirrored the differences in dietary composition, but it was also associated with the family groups. Small RNA sequencing followed by RT‐qPCR identified 12 differentially expressed microRNAs (DE miRNAs), with expression associated with family groups (miR‐146 family members, miR‐200b, miR‐214, miR‐221, miR‐125, miR‐135, miR‐137, miR_nov_1), diets (miR‐203, miR‐462) or both conditions. All the conserved DE miRNAs have been reported as associated with lipid metabolism in other vertebrates. *In silico* predictions revealed 37 lipid metabolism pathway genes, including desaturases, transcription factors and key enzymes in the synthesis pathways as putative targets (e.g., *srebp‐1* and *2*, *Δ6fad_b* and *c*, *hmdh*, *elovl4* and *5b*, *cdc42*). RT‐qPCR analysis of selected target genes showed expression changes that were associated with diet and with family groups (*d5fad*, *d6fad_a*, *srebp‐1*). There was a reciprocal difference in the abundance of ssa‐miR‐203a‐3p and *srebp‐1* in one group comparison, whereas other predicted targets did not reveal any evidence of being negatively regulated by degradation. More experimental studies are needed to validate and fully understand the predicted interactions and how the DE miRNAs may participate in the regulation of hepatic lipid metabolism.

## 1 INTRODUCTION

MicroRNAs (miRNAs) are short RNA molecules, typically 21–24 nucleotides in length, that regulate gene expression as part of the miRNA‐induced silencing complex (miRISC). The function of the miRNA is to guide the miRISC to the target transcripts by partial base pairing between the miRNA and the target mRNA [usually the 3′UTR (untranslated region)]. The binding of the miRISC to an mRNA results in the degradation of the target gene transcript or inhibition of its translation. This leads to a post‐transcriptional downregulation of the target gene protein (Chekulaeva & Filipowicz, 2009; Hausser & Zavolan, 2014; Krol *et al*., 2010). Research over the past decade has revealed several hundreds of miRNA genes, each of which has the potential to target several target transcripts. Thus, miRNAs control the expression of a large number of genes and seem to be the major post‐transcriptional regulators of cellular gene networks in vertebrates (Friedman *et al*., 2009). Studies in teleost fish have shown that miRNAs participate in the regulation of early development, apoptosis, the maintenance of tissue‐specific functions, reproduction and immune response (Andreassen & Hoyheim, 2017; Bizuayehu & Babiak, 2014; Chen *et al*., 2019). Studies on miRNAs in commercially important fish species have indicated that they also participate in the regulation of economically interesting traits like growth or food conversion (Andreassen *et al*., 2016; Mennigen, 2016).

The long‐chain polyunsaturated omega‐3 fatty acids eicosapentaenoic acid (EPA) and docosahexaenoic acid (DHA) are essential nutrients for the health of both Atlantic salmon (*Salmo salar*) and humans. These fatty acids exert a range of health benefits through their molecular, cellular and physiological actions (Bou *et al*., 2017a; Bou *et al*., 2017b; Calder, 2014). EPA and DHA are synthesized from the essential fatty acid 18:3n‐3 in a cascade of reactions consisting of elongation (catalysed by elongase2 and elongase5), desaturation (by delta 5 desaturase and delta 6 desaturase) and a final peroxisomal beta‐oxidation step (by acetyl co‐A oxidase) (Sprecher, 2000).

Factors such as diets, life stage, genotype and growth are known to influence the capacity for EPA and DHA syntheses in Atlantic salmon (Tocher *et al*., 2000; Rosenlund *et al*., 2001; Bell *et al*., 2002; Torstensen *et al*., 2005; Leaver *et al*., 2011; Thomassen *et al*., 2012). The dietary content of marine oil and vegetable oil will affect both the deposition of fatty acids in the fish liver and muscle and the synthesis of the fatty acids EPA and DHA. Marine fish oils typically contain high levels of EPA and DHA, which are devoid of vegetable oils. Vegetable oils, on the contrary, are usually high in linoleic acid (18:2n‐6) and monounsaturated fatty acids such as 18:1n‐9. Specific fatty acids of the dietary oil fraction can act inhibitory or stimulatory on the synthesis of EPA and DHA in Atlantic salmon. Feeding salmon high‐dietary levels of EPA and DHA results, *e.g*., in downregulation of gene expression of desaturases and elongases involved in omega‐3 synthesis, whereas high dietary levels of vegetable oil and low levels of EPA and DHA result in the upregulation of the expression (Kjaer *et al*., 2008; Morais *et al*., 2009; Moya‐Falon *et al*., 2005; Ruyter *et al*., 2003). The genetic background is also important for the EPA and DHA composition in salmon, and the DHA content of salmon fillet was recently identified as a highly heritable trait (*h* = 0.46) (Horn *et al*., 2018). Nonetheless, the correlation between the liver and muscle content of EPA and DHA seems to be low (Horn *et al*., 2019), indicating that the capacity for EPA and DHA syntheses in liver is less important for the levels of these fatty acids in the muscle. Various genes are involved in hepatic lipid metabolism (Morais *et al*., 2011; Torstensen *et al*., 2009). Many of these lipogenic genes are regulated by the transcription factors, sterol regulatory element binding proteins (SREBP) srebp1 and srepb2 (Minghetti *et al*., 2011). Srepb1 plays a crucial role in the regulation of fatty acid biosynthesis, whereas cholesterol biosynthesis is regulated by Srebp2. Several other genes and gene networks involved in lipid metabolism have been identified in transcriptome studies of salmon, and a connection between fatty acid accumulation, dietary lipid content and immune response has been revealed (Martinez‐Rubio *et al*., 2012; Martinez‐Rubio *et al*., 2013; Skugor *et al*., 2010).

Characterizations of miRNAs associated with lipid metabolism in teleost fish have been carried out by manipulation of dietary lipids in tilapia, rabbit fish and rainbow trout (Mennigen *et al*., 2014; Tao *et al*., 2017; Zhang *et al*., 2014). These studies have revealed smaller groups of differentially expressed miRNAs (DE miRNAs) likely to be involved in the regulation of lipid metabolism. Genes that might be the target transcripts were identified in a few cases and shown to be among the key genes in lipid metabolism gene networks. The miRNAome is well characterized in Atlantic salmon (Andreassen *et al*., 2013; Woldemariam *et al*., 2019), and several miRNAs that respond to viral infection and that are likely to regulate inflammatory response have been identified (Andreassen *et al*., 2017; Woldemariam *et al*., 2020). Nonetheless, to authors’ knowledge, there are no studies of miRNAs and their putative regulatory roles in the salmon lipid metabolism. The molecular mechanism leading to the reported dietary effect from EPA and DHA on expression of genes in the omega‐3 synthesis pathway (Kjaer *et al*., 2008; Morais *et al*., 2009; Moya‐Falon *et al*., 2005; Ruyter *et al*., 2003) could, *e.g*., involve post‐transcriptional regulation by miRNAs.

This study investigates how a diet that was either rich in fish oil and low in rapeseed oil (75FO) or low in fish oil and rich in rapeseed oil (10FO) affects the hepatic fatty acid composition in two family groups of Atlantic salmon with different capacities to produce EPA and DHA (HIGH and LOW family groups). The individual miRNA expression in the HIGH and LOW family groups fed either of the two diets (75FO/HIGH, 75FO/LOW, 10FO/HIGH or 10FO/LOW) was subsequently revealed. Comparisons between the four diet/family groups could then uncover whether a difference in dietary fatty acids (diet) and/or a difference in family background was associated with the different expression of individual miRNAs. The identification of DE miRNAs was carried out applying small‐RNA sequencing followed by RT‐qPCR. The small‐RNA Illumina sequencing and differential expression analysis of a smaller number of samples selected from all four groups was carried out to allow for identification of any Atlantic salmon miRNAs with putative regulatory functions in the lipid metabolism pathways. The miRNAs identified by this approach were subsequently validated as differentially expressed by additional RT‐qPCR analysis in the larger complete materials. Even if not pointed out as putative DE miRNAs by the small‐RNA sequencing, a small number of miRNAs known to have important roles in lipid metabolism in other vertebrates were also analysed by RT‐qPCR in the larger complete materials to confirm the negative results in the small‐RNA‐sequenced materials. The putative target genes of the DE miRNAs were predicted, and some of these predicted targets were further analysed using RT‐qPCR. Finally, the expression changes in the DE miRNAs and their predicted targets were compared to further elucidate the role(s) of individual miRNAs as regulators of lipid metabolism in Atlantic salmon.

## 2 MATERIALS AND METHODS

### 2.1 Family fish groups and feeding trial

The feeding trial was carried out at Nofima Research Station for Sustainable Aquaculture (Sunndalsøra, Norway). The experimental fish were from a Norwegian Research Council project (Towards a sustainable salmonid aquaculture – Salmon as a net producer of n‐3 fatty acids). In this project, 100 families of Atlantic salmon (*S. salar* L.) from SalmoBreed AS Elite stock were tested for their expression of the Δ6 desaturase isomer *Δ6fad_b*, and families with average high expression (HIGH) and low expression (LOW) of *Δ6fad_b* were used as parental individuals to produce new families as described in Berge *et al*. (2015). The materials used in this study are progeny (first‐generation fish) from crosses within the HIGH families (HIGH family group) and within the LOW families (LOW family group). HIGH and LOW groups with 150–204 fish per group were included in the Norwegian Research Council project, and 18 individuals from the HIGH family group and 18 individuals from the LOW family group were included in this study. The fish were fed a diet with either 10% (10FO) or 75% (75FO) fish oil for 17 weeks and increased weight from 76 ± 14 to 525 ± 75 g. All fish were individually tagged (PIT tags, passive integrated transponder, Biosonic, Seattle, USA), and fish from both family groups were equally distributed in triplicate tanks for each of the two diets, in total 12 tanks. At the end of the feeding trial, the fish were anaesthetized, and livers from nine fish per diet and family group (three fish per tank) were snap frozen in liquid nitrogen and stored at −80°C until further analysis. The fish materials, thus, consisted of four groups divided by their family background and diet: (a) 10FO/LOW, (b) 10FO/HIGH, (c) 75FO/LOW and (d) 75FO/HIGH.

The trial was performed in accordance with the national regulations for the use of animals in experiments (Ministry of Agriculture and Food, 2015). The experiment was classified as not requiring a specific licence (Commission, 2010) because the experiment was not expected to cause any distress or discomfort to the fish.

### 2.2 Fatty acid composition of the diets 10FO and 75FO

The diets were formulated to contain two different levels of fish oil, 10% fish oil (10FO) or 75% fish oil (75FO) of the oil fraction. Table 1 provides the chemical composition, whereas Table 2 provides the fatty acid composition of the two diets. The resulting fatty acid composition of the 75FO diets thereby contained a higher percentage of EPA (C20:5n‐3), docosapentaenoic acid (DPA, C22:5n‐3) and DHA (C22:6n‐3) than the 10FO. The sum of EPA and DHA in the 75FO was 22.1% (of total fatty acids) compared to 6.9% in the 10FO. The 18:3n‐3 was, nonetheless, higher in 10FO (7.5%) than in 75FO (2.8%). The 75FO had a higher percentage of saturated fatty acids (SFAs) than 10FO, constituted mainly by 14:0, 16:0 and 18:0. The percentage of monounsaturated fatty acids (MUFAs) was also higher in 10FO than in 75FO, resulting mostly from a 2.3 times higher level of 18:1n‐9 in the 10FO. The sum of n‐6 fatty acids was higher in 10FO compared to 75FO and was dominated by 18:2n‐6 (linoleic acid) that was 2.4 times higher in 10FO than in 75FO.

**TABLE 1:** Chemical composition and EPA + DHA (g per 100) of the diets

	10FO	75FO
Dry matter	92.9	93
Fat	27.6	27.1
Protein	43.3	43.9
Ash	8	8
EPA + DHA (g per 100 g)	1.7	5.4

*Note*. DHA: docosahexaenoic acid; EPA: eicosapentaenoic acid.

**TABLE 2:** Fatty acid composition (% of total fatty acids) of the diets

Fatty acid	10FO	75FO
14:0	1.5	5.7
16:0	7.3	14.1
18:0	2.6	3.1
Sum SFA^a^	12.7	24.5
16:1 n‐7	1.8	6.9
17:1 n‐7	0.3	1.1
18:1 n‐11	Not detected	1.6
18:1 n‐9	45.9	19.7
18:1 n‐7	2.3	2.9
20:1 n‐11	0.5	1.8
20:1 n‐9	1.9	1.4
22:1 n‐11	0.1	0.7
Sum MUFA^b^	53.4	37.5
18:2 n‐6	17.3	7.1
20:4 n‐6	0.2	0.8
Sum n‐6 PUFA^c^	17.7	8.4
18:3 n‐3	7.5	2.8
20:5 n‐3	4.2	12.9
22:5 n‐3	0.4	1.5
22:6 n‐3	2.7	9.2
Sum n‐3 PUFA^d^	14.9	26.8
EPA + DHA	6.9	22.1
Ratio n6/n3	2.6	0.4

*Note*. DHA: docosahexaenoic acid; EPA: eicosapentaenoic acid; MUFA: monounsaturated fatty acid; PUFA: polyunsaturated fatty acid; SFA: saturated fatty acid.

^a^ Includes 15:0, 17:0, 20:0, 22:0, 24:0.

^b^ Includes 14:1n‐5, 15:1, 16:1n‐9, 16:1n‐5, 20:1n‐7, 22:1n‐7, 22:1n‐9, 24:1n‐9.

^c^ Includes 16:2 n‐6, 18:3n‐6, 20:3n‐6, 20:2n‐6, 22:4n‐6.

^d^ Includes 16:2n‐3, 20:4n‐3, 18:4n‐3, 20:3n‐3.

### 2.3 Measurements of fatty acid composition of the liver

The fatty acid composition of the 36 liver samples (9 fish per group) was analysed by trans‐methylating the lipids re‐dissolved in chloroform using 2,2‐dimethoxypropane, methanolic HCl and benzene at room temperature, as described by Mason (Mason & Waller, 1964). The methyl esters of fatty acids were then separated in a gas chromatograph (Hewlett Packard 6890) with a split injector, SGE BPX70 capillary column (length 60 m, internal diameter 0.25 mm and thickness of the film 0.25 μm), flame ionization detector and HP Chem Station software. The carrier gas was helium. The injector and detector temperatures were 300°C. The oven temperature was raised from 50 to 170°C at a rate of 4°C min^–1^ and thereafter raised to 200°C at a rate of 0.5°C min^–1^ and finally to 300°C at a rate of 10°C min^–1^. The relative quantity of each fatty acid was determined by measuring the area under the peak in the gas chromatograph spectrum corresponding to the specific fatty acids.

### 2.4 Isolation of RNA

RNA was isolated from 36 salmon liver samples (9 fish per group) using mirVana miRNA Isolation Kit (Thermo Fisher Scientific, Waltham, MA, USA) according to the manufacturer's protocol. Concentration and purity were evaluated using a NanoDrop ND‐1000 spectrophotometer (NanoDrop Technologies,Wilmington, DE, USA) and the integrity of the RNA using an Agilent 2100 bioanalyzer/Agilent RNA 6000 Nano Kit (Agilent Technologies, Santa Clara, CA, USA). The 36 samples showed concentrations of total RNA ranging from 324 to 1200 ng μl^–1^. All samples showed 260/280 ratios of 2.0 or more. Thirty‐one samples showed 260/230 ratios above 2.0, whereas the remaining five samples ranged from 0.95 to 1.76. Downstream analysis (RT‐qPCR) did not reveal any of these five samples as outliers within their groups. The RNA concentrations as well as quality measurements for all samples are provided in Supporting File S1.

### 2.5 cDNA synthesis and miRNA expression measurements using RT‐qPCR

The miScript assays were used for cDNA synthesis and qPCR as described by the manufacturer (Qiagen, Hilden, Germany). In the cDNA synthesis, 200 ng of total RNA was reverse transcribed utilizing the miScript II RT kit. The procedure was performed according to the manufacturer's protocol. The reaction mixture consisted of 4 μl of 5x HiSpec Buffer, 2 μl of 10x Nucleics Mix, 2 μl of Reverse Transcriptase Mix, a variable amount of RNase free water and template RNA to a total volume of 20 μl for each reaction. Incubation of the RT reactions was carried out in a 2720 Thermal Cycler (Applied Biosystems, Foster City, CA, USA) at 37°C for 60 min followed by an inactivation step at 95°C for 5 min and finally holding it to cool down at 4°C. All samples were placed on ice, then instantly diluted with 200 μl of RNase free water and stored at −20°C. A universal primer (reverse primer), provided with the miScript qPCR kit, was used in combination with the authors’ custom‐designed forward primer in the qPCR assays amplifying each of the mature miRNAs. The sequence of the mature miRNAs investigated in this study was utilized to produce miRNA‐specific forward primers. All primers were purchased from Sigma‐Aldrich (Darmstadt, Germany), purified by desalt only and provided as a liquid solution of 100 μM from the manufacturer. They were diluted to 10 μM for use in each of the qPCR assays. The qPCR analysis was run on an Mx3000p (Stratagene, San Diego, CA, USA). The qPCR reaction mixture consisted of 12.5 μl 2x Quantitect Syber Green Master Mix, 2.5 μl 10x miScript Universal Primer, 2.5 μl of 10 μM forward miRNA‐specific primer, 5 μl of Rnase free water and 2.5 μl of cDNA (template). The following programme was used in qPCR: one thermal cycle at 95°C for 15 min followed by 40 cycles at 94°C for 15 s, 55°C for 30 s and 70°C for 30 s. The Mx3000p software package was used for qPCR analysis. The SYBR Green assay module includes a final melting point analysis that follows the 40 cycles of qPCR. Plots from melting point analysis were manually inspected for all the miRNA assays tested to verify that forward primers were specific. The efficiency was calculated using the LinRegPCR software (Ramakers *et al*., 2003). The primer sequences of all the miRNA‐specific primers (forward primers) used for measurements of miRNA expression using RT‐qPCR, the efficiencies of the assays and the melting curves are provided in Supporting File S2. The relative change in miRNA expression between groups was calculated using efficiency‐adjusted Ct values and the comparative Ct method (ΔΔCt method) (Schmittgen & Livak, 2008). The normalized read counts from the small‐RNA‐sequenced samples were utilized to compare the stability of ssa‐miR‐25‐3p, ssa‐miR‐92a‐3p, ssa‐miR‐181a‐3p, ssa‐miR‐455‐5p, ssa‐miR‐107‐3p and ssa‐miR‐17‐5p across all groups, an approach similar to the one applied in Johansen and Andreassen (2014) to select candidate reference miRNAs. They all showed high stability across all groups, and three of these miRNAs (ssa‐miR‐25‐3p, ssa‐miR‐92a‐3p and ssa‐miR‐181a‐3p) were analysed in all samples using RT‐qPCR. The following normfinder analysis carried out as described in Johansen and Andreassen (2014) showed that ssa‐miR‐25‐3p and ssa‐miR‐92a‐3p were the best reference gene combination with a combined stability value of 0.002. These two miRNAs were used as reference genes in the miRNA expression analysis.

Nine individuals from each group, *i.e*., 36 individuals, were analysed using RT‐qPCR. Based on the findings from the expression analysis of the small‐RNA‐sequenced samples (DESeq2 analysis, see Section 2.6), the miRNAs ssa‐miR‐146a‐5p, ssa‐miR‐146a‐3p, ssa‐miR‐146a‐3‐3p, ssa‐miR‐462b‐5p, ssa‐miR‐nov‐1‐5p, ssa‐miR‐200b‐5p, ssa‐miR‐203a‐3p, ssa‐miR‐214‐5p, ssa‐miR‐221‐5p, ssa‐miR125a‐3p, ssa‐miR‐135c‐5p, ssa‐miR‐137a‐3p and ssa‐miR‐92b‐3p were analysed using RT‐qPCR in the complete materials to validate that they were DE miRNAs. Some miRNAs have been pointed out by studies in other vertebrates as associated with lipid metabolism. Although these were not significantly different in the DESeq2 analysis, they were included in the RT‐qPCR analysis and investigated in a larger sample to rule out the possibility of type II errors (false positives) in the DESeq2 analysis. These miRNAs were ssa‐miR‐10b‐5p, ssa‐miR‐15a‐5p, ssa‐miR‐17‐5p, ssa‐miR‐21a‐5p, ssa‐miR‐26a‐5p, ssa‐miR‐27a‐3p, ssa‐miR‐27b‐3p, ssa‐miR‐30c‐5p, ssa‐miR‐30e‐5p, ssa‐miR‐30e‐3p, ssa‐miR‐33a‐3p, ssa‐miR‐122‐5p, ssa‐miR‐143‐3p, ssa‐miR‐145‐3p and ssa‐miR‐192a‐5p (Ahn *et al*., 2013; Casas‐Agustench *et al*., 2015; Chen *et al*., 2014; Fernandez‐Hernando, 2013; Karbiener *et al*., 2014; Mennigen *et al*., 2014; Sala *et al*., 2014; Shin *et al*., 2014; Smolle & Haybaeck, 2014; Soh *et al*., 2013; Sun *et al*., 2015; Yang *et al*., 2015; Zhang *et al*., 2014). One miRNA, ssa‐miR‐nov‐1‐3p, showed a significant differential expression in the DESeq2 analysis, but the RT‐qPCR assays developed for this miRNA did not pass the performance criteria (specificity, efficiency). Thus, this miRNA was not further analysed.

### 2.6 Small‐RNA sequencing and expression analysis (DESeq2)

The library construction was performed at the Norwegian Genomics Consortium's genomics core facility. The Illumina NEBnext Small RNA Library Preparation Kit (New England Biolabs, Inc., Ipswich, MA, USA) was used for the library preparations as described by the manufacturer with 1 μg of total RNA input. After adapter ligation and cDNA synthesis the products were purified on a gel, and the fractions between 145 and 160 bp were used for sequencing. Twelve small‐RNA libraries were constructed from 12 samples (3 samples from each of the 4 groups: 10FO/LOW, 10FO/HIGH, 75FO/LOW and 75FO/HIGH). The libraries were successfully subjected to high‐throughput sequencing using Illumina Genome Analyser IIx sequencing platform as described in Andreassen *et al*. (2017). FastQC toolkit was used to assess reads quality (http://www.bioinformatics.babraham.ac.uk/projects/fastqc). Cutadapt (Martin, 2011) was used for trimming of adapter sequences from raw sequence reads and for removing adapter‐only sequences (5′TGGAATTCTCGGGTGCCAAGGAACTCCAGTCAC 3′). Finally, an additional filtering of reads outside of the 18–25 nucleotide range was applied on all samples.

Next, the reads from each of the 12 samples were aligned to a reference miRNAome that consisted of all known *S. salar* mature miRNAs (Andreassen *et al*., 2013; Woldemariam *et al*., 2019). The reads mapping with edit distance 1 or less to the mature reference sequences were counted. DE miRNAs were identified using the DESeq2 package (Anders & Huber, 2010). Rows with fewer than two reads for each condition were discarded from the analysis. The four groups were compared, and a threshold of *P*‐adjusted value <0.1 (adjusted according to Benjamini–Hochberg procedure) was applied to report the putative DE miRNAs.

### 2.7 *In silico* predictions of target transcripts

Target gene predictions were carried out using RNAhybrid. The analysis was performed with conditions of helix constraint 2–8 and no G:U in seed, allowing only target genes that had perfect “seed” matches to be detected (Rehmsmeier *et al*., 2004). The minimum free energy threshold for RNA hybrids was set to −18 kcal mol^–1^ to retrieve results (target site matches) from RNA hybrids that had a high stability (Peterson *et al*., 2014). The 3′UTR sequences from all *S. salar* transcripts in Genbank (Non‐redundant mRNA, NM entries in RefSeq, NCBI) were used as an input in the *in silico* analysis.

Gene ontology (GO) annotations for the predicted target genes (biological process and molecular function) were received for each of the target genes using UniProt database (http://www.uniprot.org/uploadlists/). These GO annotations were used to identify the sub‐set of predicted targets that were associated with lipid metabolism.

### 2.8 Gene expression analysis of predicted target genes

cDNA was synthesized from 1000 ng of total RNA using TaqMan Reverse Transcription Reagents (Applied Biosystems) in a 20 μl reaction. The reaction mixture consisted of 1x TaqMan RT Buffer, 1.75 mM MgCl₂, 0.5 mM deoxynucleotide triphosphate mixture, 2.5 μM Oligo d(T)_16_, 1 U μl^–1^ RNase inhibitor and 2.5 U μl^–1^ Reverse Transcriptase. The reaction was run in a Verti 96‐well thermal cycler (Thermo Fisher Scientific) with the following conditions: 25°C for 10 min, 48°C for 60 min and 95°C for 5 min. The PCR mixture consisted of 4 μl of cDNA (1:10 dilution), 5 μl of LightCycler480 SYBR Green I Master (Roche Diagnostics Gmbh, Mannheim, Germany) and 0.5 μM forward and reverse primers (Thermo Fisher Scientific). The reaction was run in a LightCycler480 (Roche Diagnostics Gmbh) with the following conditions: 95°C for 5 min, 45 cycles of 95°C for 15 s and 60°C for 1 min. A melting curve analysis (95°C for 5 s, 65°C for 1 min, increasing temperature up to 97°C and finally cooling down) was performed to confirm the presence of only one amplified product (see Supporting File S3 for pictures of melting curve analysis and references). The stability of the reference genes (*ef1a*, *rpol2* and *eif3*) was evaluated using RefFinder (https://github.com/fulxie/RefFinder), which integrates the computational programmes geNorm, NormFinder and BestKeeper and the comparative ΔΔCT method. According to the RefFinder results, eukaryotic transcription initiation factor 3 (*eif3*) was ranked as the most stable reference gene (Supporting File S3). Primer efficiency was evaluated by making a standard curve (consisting of six dilutions of cDNA), and calculation of the amplification efficiency was performed using the software included in LightCycler480 (see Supporting File S3 for primer efficiency and references for primer efficiencies evaluated in previous studies, primer sequences and GenBank accession numbers).

The expression of eight genes from lipid metabolism gene networks was measured in all individuals using RT‐qPCR. These were delta 5 fatty acid desaturase (*Δ5fad*), delta 6 fatty acid desaturase‐a (*Δ6fad_a*), delta 6 fatty acid desaturase‐b (*Δ6fad_b*) and delta 6 fatty acid desaturase‐c (*Δ6fad_c*) that are enzymes involved in the omega‐3 fatty acid synthesis pathway; sterol regulatory element binding proteins 1 and 2 (*srebp1*, *srebp2*) that are transcription factors; carnitine palmitoyltransferase I (*cpt1*) and acyl‐CoA oxidase 1 (*acox*) that are involved in fatty acid oxidation. The relative gene expression level was calculated according to the ΔΔCt method adjusting for differences in primer efficiency (Pfaffl, 2004).

### 2.9 Statistical methods applied to test group differences in fatty acid composition, miRNA expression and mRNA gene expression

The effects of diet and genetic background and the interaction between the main factors for fatty acid composition, miRNA expression and gene expression data were assessed using a two‐way factorial design. Data were statistically analysed using the general linear model (GLM) procedure in SAS9.4 software (SAS Institute, Cary, NC, USA). The differences in miRNA expression and mRNA gene expression were ranked using Duncan's multiple range test. Statistical analyses were conducted using the software package UNISTAT (Unistat Ltd, London, England).

## 3 RESULTS

### 3.1 Hepatic fatty acid composition is affected by diet, genetic background and their interaction

The analysis of the fatty acid composition in liver showed a significant effect of both diets and family background on multiple fatty acids, demonstrating that the experimental conditions had affected the hepatic lipid metabolism in the four groups compared. The hepatic fatty acid composition largely mirrored the diet composition when comparing 10FO and 75FO groups, but comparisons within the same diet conditions showed that there were also significant differences due to family background (LOW/HIGH). The complete results from measurements of fatty acid composition along with results from two‐way ANOVA tests for significant differences are provided in Table 3.

**TABLE 3:** Hepatic fatty acid composition

	10FO		75FO		Two‐way ANOVA		
	LOW	HIGH	LOW	HIGH	Diet	Genetics	Interaction
14:0	1.0 ± 0.0	1.0 ± 0.0	1.8 ± 0.3	1.8 ± 0.1	<0.001	NS	NS
16:0	5.7 ± 0.4	6.6 ± 0.4	11.0 ± 0.4	14.9 ± 0.7	<0.0001	<0.001	<0.05
18:0	5.0 ± 0.2	4.8 ± 0.1	6.5 ± 0.1	6.0 ± 0.3	<0.0001	0.05	NS
∑SFA^a^	12.3 ± 0.5	13.0 ± 0.5	20.1 ± 0.6	23.5 ± 0.5	<0.0001	<0.05	<0.05
16:1n‐9	1.8 ± 0.2	1.5 ± 0.1	3.4 ± 0.2	2.2 ± 0.3	<0.0001	<0.05	NS
18:1n‐9	48.3 ± 0.3	43.9 ± 1.4	24.3 ± 0.5	14.5 ± 1.7	<0.0001	<0.001	NS
18:1n‐7	3.5 ± 0.1	3.3 ± 0.1	4.2 ± 0.2	3.1 ± 0.2	NS	<0.001	<0.05
20:1n‐11	0.2 ± 0.0	0.2 ± 0.0	0.6 ± 0.0	0.4 ± 0.1	<0.0001	<0.05	<0.05
20:1n‐9	6.0 ± 0.0	5.9 ± 0.2	2.8 ± 0.1	1.9 ± 0.2	<0.0001	<0.05	0.05
22:1n‐7	0.6 ± 0.0	0.6 ± 0.0	1.1 ± 0.1	0.8 ± 0.0	<0.0001	<0.05	<0.05
24:1n‐9	0.3 ± 0.0	0.5 ± 0.0	0.9 ± 0.0	1.4 ± 0.1	<0.0001	<0.0001	<0.05
∑MUFA^b^	61.8 ± 0.2	56.8 ± 1.8	39.0 ± 0.8	25.4 ± 2.4	<0.0001	<0.0001	<0.05
18:2n‐6	10.2 ± 0.4	10.3 ± 0.2	4.2 ± 0.3	3.1 ± 0.2	<0.0001	NS	NS
20:2n‐6	2.5 ± 0.1	2.6 ± 0.0	1.1 ± 0.0	0.9 ± 0.0	<0.0001	NS	<0.05
20:3n‐6	1.2 ± 0.1	1.2 ± 0.1	0.4 ± 0.0	0.4 ± 0.0	<0.0001	NS	NS
20:4n‐6	0.6 ± 0.1	0.9 ± 0.1	2.2 ± 0.1	3.2 ± 0.3	<0.0001	<0.05	NS
∑n‐6 PUFA^c^	14.9 ± 0.6	15.2 ± 0.2	8.4 ± 0.4	8.0 ± 0.2	<0.0001	NS	NS
18:3n‐3	2.5 ± 0.1	2.5 ± 0.1	1.1 ± 0.1	0.8 ± 0.1	<0.0001	NS	NS
20:3n‐3	0.6 ± 0.0	0.6 ± 0.0	0.3 ± 0.0	0.3 ± 0.0	<0.0001	NS	NS
20:5n‐3	1.3 ± 0.1	1.7 ± 0.1	7.0 ± 0.4	8.6 ± 0.3	<0.0001	<0.05	NS
22:5n‐3	0.3 ± 0.0	0.4 ± 0.0	2.6 ± 0.1	2.5 ± 0.1	<0.0001	NS	NS
22:6n‐3	4.4 ± 0.2	7.9 ± 1.1	18.2 ± 0.6	27.8 ± 1.6	<0.0001	<0.001	<0.05
∑n‐3 PUFA^d^	9.1 ± 0.2	13.1 ± 1.2	29.3 ± 0.9	39.9 ± 1.8	<0.0001	<0.001	<0.05
EPA + DHA	5.7 ± 0.2	9.6 ± 1.3	25.2 ± 0.9	36.4 ± 1.9	<0.0001	<0.001	<0.05
Ratio n‐6/n‐3	5.7 ± 0.2	9.6 ± 1.3	25.2 ± 0.9	36.4 ± 1.9	<0.0001	<0.001	<0.05

*Note*. Fatty acid composition in liver (% of total fatty acids) of salmon fed 10FO or 75FO diets in LOW and HIGH family groups.

The two‐way ANOVA statistics are shown with *P*‐values for the effect of diets (10FO and 75FO), family groups (LOW and HIGH family groups) and the interaction between diet and family groups. NS: not significant (*P* > 0.05). Data are shown as mean ± s.e. (*n* = 3).

DHA: docosahexaenoic acid; EPA: eicosapentaenoic acid; MUFA: monounsaturated fatty acid; PUFA: polyunsaturated fatty acid; SFA: saturated fatty acid.

^a^ Includes 15:0, 17:0, 20:0, 22:0, 24:0.

^b^ Includes 14:1n‐5, 16:1n‐7, 16:1n‐5, 17:1n‐7, 19:1, 20:1n‐7, 22:1n‐11, 22:1n‐9.

^c^ Includes 16:2n‐6, 18:3n‐6.

^d^ Includes 20:4n‐3.

The relative liver composition of DHA, the sum of the polyunsaturated n‐3 fatty acids (PUFA) and the sum of EPA and DHA, was significantly affected by diets, family groups and their interaction. The relative EPA levels (20:5n‐3) were 1.2–1.3 times higher in the HIGH groups than in the LOW groups showing EPA levels of 1.7% (10FO/HIGH) and 8.6% (75FO/HIGH). The relative DHA levels were 1.5–1.8 times higher in the HIGH groups than in the LOW groups with DHA levels of 7.9% (10FO/HIGH) and 27.8% (75FO/HIGH). Nevertheless, the increased content of fish oil in the diet of the 75FO groups resulted in a much higher percentage of PUFA (including EPA and DHA) in the 75FO group than in the 10FO group (Table 3).

The diet, family background and their interaction also affected the relative level of the sum of SFAs (∑SFA, Table 3), with a higher percentage in the 75FO group (20.1%–23.5%) than in the 10FO group (12.3%–13.0%) and in the HIGH group compared to the LOW group within the 75FO group.

The percentage of MUFAs (∑MUFA, Table 3) was affected by both diets, family groups and their interaction, with the 10FO groups (56.8% and 61.8%) showing 1.6–2.2 times higher level than the 75FO groups (39.0% and 25.4%). The HIGH groups had a significantly lower relative level of MUFAs compared to the LOW groups (0.7–0.9 times). Within the 75FO group, the HIGH group had a lower liver level of several fatty acids such as 16:1n‐9, 18:1n‐9, 18:1n‐7 and 20:1n‐9 than the LOW group.

### 3.2 Small‐RNA sequencing followed by RT‐qPCR analysis identified 12 DE miRNAs

Small‐RNA sequencing followed by RT‐qPCR analysis was applied to identify DE miRNAs when comparing the salmon groups fed 10FO or 75FO and the two family groups HIGH and LOW. Three samples from each group were small‐RNA sequenced. The descriptive data from the 12 samples used in the small‐RNA sequencing along with the results from FASTQC analysis are provided in Supporting File S4. All samples were successfully sequenced with per base Phred quality scores of 32 or more. The percentage of reads mapped as miRNAs in the size‐filtered and adapter‐processed samples ranged from 83.1% to 87.5%. The expression of all the known salmon miRNAs was investigated by differential expression analysis (DESeq2) (Supporting File S5). The results from DESeq2 analysis pointed out 14 putative DE miRNAs. Thirteen of these were further analysed by RT‐qPCR, whereas one, ssa‐miR‐nov‐1‐3p, was not further validated as the RT‐qPCR assay failed.

Two‐way ANOVA analysis of the results from RT‐qPCR revealed that 12 of the 13 miRNAs investigated (ssa‐miR146a‐3‐3p, ssa‐miR‐146a‐3p, ssa‐miR‐146a‐5p, ssa‐miR‐462b‐5p, ssa‐miR‐200b‐5p, ssa‐miR‐203a‐3p, ssa‐miR‐214‐5p, ssa‐miR‐221‐5p, ssa‐miR‐125a‐3p, ssa‐miR‐137a‐3p, ssa‐miR‐135c‐5p and ssa‐miR‐nov‐1‐5p) showed significant differences associated with diet (10FO/75FO), family group (HIGH/LOW) or both. The results from the two‐way ANOVA are provided in Table 4.

**TABLE 4:** Comparison of miRNA expression in diet and family groups

	10FO		10FO		75FO		75FO		Two‐way ANOVA		
	LOW	s.d.	HIGH	s.d.	LOW	s.d.	HIGH	s.d.	Diet	Family	Interaction
miR‐146a‐3‐3p	0.00^b^ ± 0.09		−0.35^b^ ± 0.20		−0.42^b^ ± 0.17		–1.13^a^ ± 0.26		0.004	0.009	0.37
miR‐146‐a‐3p	0.00^c^ ± 0.16		−0.58^ab^ ± 0.17		−0.08^bc^ ± 0.27		−0.85^a^ ± 0.14		0.37	0.001	0.60
miR‐146a‐5p	0.00^b^ ± 0.16		−0.49^b^ ± 0.21		−0.31^b^ ± 0.21		–1.19^a^ ± 0.14		0.01	0.0007	0.30
miR‐462b‐5p	0.00^ab^ ± 0.07		0.10^b^ ± 0.14		−0.30^a^ ± 0.07		−0.12^ab^ ± 0.10		0.02	0.17	0.71
miR‐203a‐3p	0.00^ab^ ± 0.17		−0.18^a^ ± 0.16		0.36^b^ ± 0.07		0.34^b^ ± 0.14		0.003	0.48	0.54
miR‐200b‐5p	0.00^ab^ ± 0.17		0.36^b^ ± 0.22		−0.49^a^ ± 0.15		0.17^b^ ± 0.26		0.11	0.02	0.46
miR‐214‐5p	0.00^a^ ± 0.22		1.25^b^ ± 0.22		‐0.52^a^ ± 0.27		0.93^b^ ± 0.24		0.09	<0.0001	0.69
miR‐221‐5p	0.00^a^ ± 0.13		1.08^b^ ± 0.14		0.10^a^ ± 0.25		0.54^ab^ ± 0.18		0.25	0.0003	0.10
miR‐125a‐3p	0.00^a^ ± 0.21		1.07^b^ ± 0.20		‐0.08^a^ ± 0.36		0.94^b^ ± 0.28		0.69	0.0005	0.92
miR‐135c‐5p	0.00 ± 0.12		−0.39 ± 0.40		0.13 ± 0.15		−0.45 ± 0.15		0.88	0.05	0.67
miR‐137a‐3p	0.00^ab^ ± 0.58		−1.58^a^ ± 0.31		0.62^b^ ± 0.91		−1.14^a^ ± 0.25		0.36	0.007	0.88
miR‐nov‐1‐5p	0.00^b^ ± 0.31		1.45^c^ ± 0.31		‐1.66^a^ ± 0.25		0.99^c^ ± 0.15		0.0003	<0.0001	0.03

*Note*. All changes are relative to the group 10FO/LOW which is set as 0.00. Significant difference in expression between groups is indicated by different letters in superscript.

Diet: *P*‐values for comparisons of diet (10FO *vs*. 75FO).

Family: *P*‐values for comparisons of family groups selected for LOW or HIGH desaturase activity.

Interaction shows the *P*‐values for combined effect of diet and family selection for desaturase.

Despite being associated with lipid metabolism in other vertebrates, 16 miRNAs (ssa‐miR‐10b‐5p, ssa‐miR‐15a‐5p, ssa‐miR‐17‐5p, ssa‐miR‐21a‐5p, ssa‐miR‐26a‐5p, ssa‐miR‐27a‐3p, ssa‐miR‐27b‐3p, ssa‐miR‐30c‐5p, ssa‐miR‐30e‐5p, ssa‐miR‐33a‐3p, ssa‐miR‐33b‐3p, ssa‐miR‐92b‐3p, ssa‐miR‐122‐5p, ssa‐miR‐143‐3p, ssa‐miR‐145‐3p and ssa‐miR‐192a‐5p) did not reveal any differences when analysed by small‐RNA sequencing. Neither was their expression associated with the conditions investigated when analysed by RT‐qPCR in the complete materials (Ahn *et al*., 2013; Casas‐Agustench *et al*., 2015; Chen *et al*., 2014; Fernandez‐Hernando, 2013; Karbiener *et al*., 2014; Mennigen *et al*., 2014; Sala *et al*., 2014; Shin *et al*., 2014; Smolle & Haybaeck, 2014; Soh *et al*., 2013; Sun *et al*., 2015; Yang *et al*., 2015; Zhang *et al*., 2014).

The two‐way ANOVA analysis (Table 4) showed that the miR‐146 family (ssa‐miR‐146a‐3‐3p, ssa‐miR‐146a‐3p and ssa‐miR‐146a‐5p) was associated with expression differences between family groups, whereas miR‐146a‐3‐3p and ssa‐miR‐146a‐5p were differentially expressed when comparing the diet groups. The family group comparisons showed a relative decreased expression in the HIGH groups compared to the LOW groups, whereas in the diet comparison there was a relative decrease in expression in the 75FO groups when compared to the 10FO groups. The expression of the two miRNAs ssa‐miR‐462b‐5p and ssa‐miR‐203a‐3p was significantly associated only with diet. ssa‐miR‐462b‐5p showed a relative decrease in the 75FO groups, whereas ssa‐miR‐203a‐3p revealed a relative increase in the same groups.

Six miRNAs showed relative differences associated with only family groups (ssa‐miR‐200b‐5p, ssa‐miR‐214‐5p, ssa‐miR‐221‐5p, ssa‐miR‐125a‐3p, ssa‐miR‐135c‐5p and ssa‐miR‐137a‐3p). Whereas ssa‐miR‐135c‐5p and ssa‐miR‐137a‐3p showed a relative decrease in the HIGH groups, the other four miRNAs showed relative increases in the HIGH groups. The novel Atlantic salmon miRNA (ssa‐miR‐nov‐1‐5p) showed an expression associated with both diet and family background. This was also the only miRNA that revealed interaction between the two conditions. This miRNA showed a relative increase in HIGH *vs*. LOW family groups although it showed a relative decrease in the 75FO *vs*. 10FO diet groups.

### 3.3 *In silico* analysis predicted 37 lipid metabolism genes as putative target genes

The putative target genes of the DE miRNAs were predicted using the 3′UTR sequences of all *S. salar* transcript sequences in GenBank as input (see Section 2). This analysis showed 1069 predicted target genes for the 12 DE miRNAs, ranging from 20 matches by ssa‐miR‐125a‐3p to 167 matches by ssa‐miR‐203a‐3p and ssa‐miR‐nov1‐5p (data not shown).

The UniProt database (GO annotations) was used to identify a sub‐set of target genes with functions in the lipid metabolism gene pathways. One of the DE miRNAs, ssa‐miR‐200b‐5p, did not reveal any matches to such lipid metabolism genes. The remaining 11 DE miRNAs showed one or more matches to 37 lipid metabolism genes. The target genes along with the DE miRNAs predicted to target their 3′UTRs are provided in Table 5. Supporting File S6 also shows GO annotations and GenBank accession numbers.

**TABLE 5:** Predicted target genes that are part of lipid metabolism gene networks

Gene	miRNA
Peroxisomal acyl‐coenzyme A oxidase 1 (*acox1*)	miR‐146a‐3p or 3‐3p, miR‐137a‐3p, miR‐203a‐3p
Acyl‐CoA synthetase long‐chain family member 1 (*acsl1*)	miR‐146a‐3p, miR‐146a‐3‐3p, miR‐135c‐5p
Lysosomal acid lipase/cholesteryl ester hydrolase (*lich*)	miR‐nov‐1‐5p, miR‐135c‐5p, miR‐137a‐3p
Acyl‐protein thioesterase 1 (*lypa1*)	miR‐146a‐3p, miR‐146a‐3‐3p, miR‐214‐5p
*S*‐Acyl fatty acid synthase thioesterase, medium chain (*sast*)	miR‐137a‐3p, miR‐203a‐3p
Fumarylacetoacetate hydrolase domain containing 1 (*fahd1*)	miR‐nov‐1‐5p, miR‐221‐5p
Acyl‐coenzyme A thioesterase 5 (*acot5*)	miR‐146a‐3p, miR‐146a‐3‐3p
Very long‐chain acyl‐CoA synthetase (*s27a2*)	miR‐146a‐3p, miR‐146a‐3‐3p
Retinol‐binding protein II, cellular (*ret2*)	miR‐146a‐5p, miR‐135c‐5p
Apolipoprotein D (*apod*)	miR‐146a‐5p, miR‐135c‐5p
3‐Hydroxy‐3‐methylglutaryl‐coenzyme A reductase (*hmdh*)	miR‐135c‐5p, miR‐137a‐3p
Cell division control protein 42 (*cdc42*)	miR‐203a‐3p, miR‐137a‐3p
Solute carrier family 25 member 28 (*slc25a28*)	miR‐203a‐3p, miR‐137a‐3p
Sterol regulatory element binding transcription factor 2 (*srebp2*)	miR‐nov‐1‐5p
Delta‐6 fatty acyl desaturase (*d6fad‐c*)	miR‐nov‐1‐5p
Delta‐6 fatty acyl desaturase (*d6fad‐b*)	miR‐nov‐1‐5p
Delta‐5 fatty acyl desaturase (*fadsd5*)	miR‐462b‐5p
Fatty acyl‐CoA reductase 1 (*facr1*)	miR‐221‐5p
Elongation of very long‐chain fatty acids‐like 4 (*elovl4*)	miR‐221‐5p
Apolipoprotein F (*apof*)	miR‐221‐5p
Serine incorporator 1 (*serc1*)	miR‐221‐5p
ORM1‐like protein 1 (*orml1*)	miR‐221‐5p
Lipase member H (*liph*)	miR‐221‐5p
Acyl‐CoA dehydrogenase family member 11 (*acad11*)	miR‐214‐5p
Sterol regulatory element binding transcription factor 1 (*srebp1*)	miR‐203a‐3p
Lathosterol oxidase (*sc5d*)	miR‐203a‐3p
AP‐2 complex subunit mu‐1 (*ap2m1*)	miR‐203a‐3p
Carnitine *O*‐palmitoyltransferase 1 (*cpt1*)	miR‐203a‐3p
Phospholipid scramblase 2 (*pls2*)	miR‐146a‐5p
Phospholipase C delta 4 (*plcd4*)	miR‐146a‐3p
StAR‐related lipid transfer protein 5 (*star5*)	miR‐135c‐5p
Polyunsaturated fatty acid elongase elovl5b (*LOC100192340*)	miR‐135c‐5p
Proteinase‐activated receptor‐2a (*par‐2*)	miR‐135c‐5p
Probable palmitoyltransferase ZDHHC11 (*zdh11*)	miR‐135c‐5p
Phosphatidylcholine transfer protein (*ppct*)	miR‐135c‐5p
Lipolysis‐stimulated lipoprotein receptor (*lsr*)	miR‐125a‐3p
Cardiolipin synthase 1 (*crls1*)	miR‐125a‐3p

Acyl‐CoA oxidase 1 (*acox1*) was predicted as target for four DE miRNAs, whereas acyl‐CoA synthetase long‐chain family member 1 *(acsl1*), lysosomal acid lipase/cholesteryl ester hydrolase (*lich*) and acyl‐protein thioesterase (*lypa1)* were predicted as target for three DE miRNAs. Each of nine other genes (including *acot5*, *srebp2* and *s27a2*) showed target site matches for two DE miRNAs. The remaining genes, including three desaturases (*d6fad‐c*, *d6fad‐b* and *d5fad*), were targeted by a single DE miRNA. Among other genes that were predicted as targets were the two key lipid metabolism enzymes: elongation of very long‐chain fatty acids‐like 4 (*elovl4*) targeted by ssa‐miR‐221‐5p and polyunsaturated fatty acid elongase (*elovl5_b*) targeted by ssa‐miR‐135c‐5p. Both of these enzymes are essential for the synthesis of omega‐3 fatty acids.

On the contrary, each of the DE miRNAs was in most cases predicted to target several genes. Ssa‐miR‐135c‐5p was, *e.g*., predicted to target nine genes, whereas ssa‐miR‐137a‐3p, ssa‐miR‐203a‐3p and ssa‐miR‐221‐5p were predicted to target six genes each.

### 3.4 Gene expression changes in predicted target genes and miRNA/target comparisons

The seven predicted target genes *acox1*, *cpt1*, *srebp‐1*, *srebp‐2*, *Δ6fad_b*, *Δ6fad_c* and *Δ5fad* and the ∆6 desaturase gene *Δ6fad_a* were further analysed by RT‐qPCR in the complete materials. These are key genes involved in omega‐3 fatty acid synthesis (*Δ5fad*, *Δ6fad_a*, *Δ6fad_b*, *Δ6fad_c*), regulation of lipid synthesis as transcription factors (*srebp1*, *srebp2*) or lipid degradation (oxidation) (*cpt1*, *acox*). The two genes *acox* and *cpt1* did not reveal any significant changes when groups were compared, whereas the effect of diet on *Δ6fad_c* was on the borderline of significance (*P* = 0.07). In the remaining five genes, there was at least one group comparison that showed a significant difference. The results from the two‐way ANOVA analysis of these genes are summarized in Table 6.

**TABLE 6:** Comparison of hepatic gene expression of predicted target genes

	10FO		75FO		Two‐way ANOVA		
	LOW	HIGH	LOW	HIGH	Diet	Family	Interaction
d5fad	0.0 ± 0.1^a^	−0.4 ± 0.1^ab^	−0.7 ± 0.2^b^	−1.3 ± 0.2^c^	<0.0001	<0.002	0.63
d6fad_a	0.0 ± 0.2^a^	−0.2 ± 0.2^ab^	−0.6 ± 0.2^b^	−1.3 ± 0.1^c^	<0.0001	0.03	0.15
d6fad_b	0.0 ± 0.3^a^	0.1 ± 0.2^a^	−0.8 ± 0.2^b^	−1.0 ± 0.3^b^	<0.0007	0.86	0.48
d6fad_c	0.0 ± 0.1	0.0 ± 0.3	−0.5 ± 0.3	−0.6 ± 0.4	0.07	0.92	0.84
acox	0.0 ± 0.2	−0.2 ± 0.1	−0.5 ± 0.2	−0.2 ± 0.2	0.31	0.85	0.18
cpt1	0.0 ± 0.1	0.1 ± 0.1	0.0 ± 0.1	0.0 ± 0.1	0.25	0.39	0.33
srebp‐1	0.0 ± 0.2^ab^	0.6 ± 0.4^a^	−0.6 ± 0.2^b^	0.0 ± 0.4^ab^	0.08	0.05	0.97
srebp‐2	0.0 ± 0.2^ab^	0.1 ± 0.1^a^	−0.3 ± 0.2^ab^	−0.5 ± 0.1^b^	0.01	0.84	0.49

*Note*. All changes are relative to the group 10FO/LOW which is set as 0.0. Significant difference in expression between groups is indicated by different letters in superscript.

Diet: *P*‐values for comparisons of diet (10FO *vs*. 75FO).

Family: *P*‐values for comparisons of family groups selected for LOW or HIGH desaturase activity.

Interaction shows the *P*‐values for combined effect of diet and family selection for desaturase.

The ANOVA analysis showed a highly significant effect of the diet on the expression of the three genes *Δ5fad*, *Δ6fad_a* and *Δ6fad_b*, with decreased expression in 75FO compared to 10FO. A similar trend towards a dietary effect on gene expression was observed in *Δ6fad_c* (towards a decrease in 75FO groups). A significant effect of family background (HIGH *vs*. LOW groups) was also detected on the gene expression of *Δ5fad* and *Δ6fad_a*. When the two family groups were fed 75FO, the gene expression of *Δ5fad* and *Δ6fad_a* was downregulated in HIGH compared to LOW.

There was a significant effect of family background on the gene expression of sterol regulatory element binding transcription factor 1 (*srebp1*) with a relative increase in HIGH compared to LOW and a strong trend towards a dietary effect. The expression of *srebp2* was affected only by the diet with a downregulation in fish fed 75FO *vs*. 10FO in the HIGH group.

The revealed differences in the expression of some of the target genes allowed for a comparison of the abundance of the miRNAs and their predicted targets. Sterol regulatory element binding transcription factor 1 (*srebp‐1*) was predicted as target for ssa‐miR‐203a‐3p. The results showed that there was a relative decrease in *srebp‐1* in the 75FO diet group (significant in 10FO/HIGH *vs*. 75FO/LOW, Table 6). In accordance with *srebp‐1* being negatively regulated (degraded) by an ssa‐miR‐203a‐3p‐guided RISC, this miRNA showed a relative increase in expression when same groups were compared (Table 4). Sterol regulatory element binding transcription factor 2 (*srebp2*) was predicted as target for ssa‐miR‐nov‐1‐5p. Nevertheless, both the miRNA and the predicted target showed expression changes in the same direction (10FO/HIGH *vs*. 75FO/HIGH). Both *Δ6fad_b* and *Δ6fad_c* were also predicted as target for ssa‐miR‐nov‐1‐5p. These genes showed a lower expression in the 10FO/LOW *vs*. 75FO/LOW comparisons, which was significant in *Δ6fad_b* (Table 6). Nevertheless, also in this case the DE miRNA showed a change in the same direction (decrease, Table 4). The gene *Δ5fad* was predicted as target for ssa‐miR‐462b‐5p, but also in this case, both the gene and the miRNA expression changed in the same direction in group comparisons (Tables 4 and 6). Peroxisomal acyl‐coenzyme A oxidase 1 (*acox1*) and carnitine‐*o*‐palmitoyltransferase 1 (*cp1*) were also among the predicted targets. Nonetheless, they did not reveal any significant expression changes in their mRNAs (Table 6).

## 4 DISCUSSION

### 4.1 Diet, family selection, target gene expression and fatty acid composition

The reported feeding trial found that both diets and family background affected the fatty acid composition of the liver. The 75FO diet group showed much higher percentages of EPA and DHA than the 10FO diet group. Nonetheless, comparisons within both dietary groups demonstrated that the family background also contributed to the differences. The two‐way ANOVA tests also revealed a significant interaction between family groups and diets, *e.g*., the level of DHA and EPA + DHA in the liver (Table 3).

A higher inclusion level of fish oil, and therefore EPA and DHA, in the 75FO diet induced downregulation of all the desaturases, except for *d6fad_c*, compared to the 10FO diet high in rapeseed oil (Table 6). This is in agreement with findings in other studies reporting that there is a reduced synthesis capacity of EPA and DHA and a decreased gene expression of desaturases, elongases and sterol regulatory element binding protein 1 (*srepb1*) when Atlantic salmon is fed high‐dietary levels of fish oils, whereas high‐dietary vegetable oil levels resulted in the opposite effect (Kjaer *et al*., 2008; Morais *et al*., 2009; Moya‐Falon *et al*., 2005; Ruyter *et al*., 2003). Even though the 10FO groups had a higher gene expression level of the desaturases than the 75FO groups, the expression differences in *srepb1*, which regulate desaturase expression, were less affected by diet (*P* = 0.08) in the present study. Also, despite a selection of parental individuals into HIGH and LOW groups based on *d6fad_b* expression, the differences in the gene expression that were observed between the two family groups were not significant for this desaturase in the materials. The first‐generation offspring did not show any obvious added effect on *d6fad_b* expression from crossing the parental individuals from families with high‐average *d6fad_b* expression. This could indicate that there is genetic variation in several genes within the parental generation that affects the *d6fad_b* expression and not necessarily variation in the same genes that led to the average high expression in the different parental HIGH families. On the contrary, both the EPA/DHA percentage and other key genes like *d5fad*, *d6fad_a* and *srebp‐1* did reveal significant differences associated with family background.

Studies have shown that other fatty acids also influence omega‐3 synthesis (and desaturase expression). The synthesis capacity for EPA and DHA is partly regulated by the availability of the precursors 18:3n‐3. The increased expression of genes of the omega‐3 synthesis pathway was, *e.g*., observed in Atlantic salmon fed a vegetable oil high in 18:3n‐3 compared to salmon fed a fish oil low in 18:3n‐3 (Gillard *et al*., 2018). Another fatty acid, 18:1n‐9, which is the most abundant fatty acid in rapeseed oil and is found at high levels in the 10FO diet, has also been shown to stimulate the gene expression of the *Δ5*‐ and *Δ6fads* in salmon hepatocytes (Kjaer *et al*., 2016). Compared to the 75FO diet, the rapeseed oil–based 10FO diet had higher levels of both 18:3n‐3 and 18:1n‐9. In accordance with other studies (Gillard *et al*., 2018), the gene expression of the *Δ5*‐ and *Δ6fads* was higher in the 10FO compared to the 75FO (Table 6). Nonetheless, as revealed in the measurements of fatty acid composition (Table 3), the increased availability of 18:3n‐3 and 18:1n‐9 in the 10FO diet did not stimulate omega‐3 synthesis to produce EPA and DHA to levels gained from the 75FO diet. Atlantic salmon has all the enzymes for EPA and DHA synthesis, but, as demonstrated here, the capacity is limited. Zheng *et al*. (2009) showed that the molecular mechanism that leads to EPA suppressing the omega‐3 synthesis in salmon cells could be by suppressing the activity of the Δ6fad promoter. Nonetheless, five of the miRNAs identified as differentially expressed in this study could, as discussed in Section 4.2, also be involved in such diet‐triggered regulation by targeting key lipid metabolism genes.

The transcription factor Srebp‐2 predominantly regulates cholesterol biosynthesis, and its expression is increased by the depletion of cholesterol (Horton *et al*., 2002). A suppression of the cholesterol synthesis pathway was also observed in Atlantic salmon fed a cholesterol‐supplemented diet (Kortner *et al*., 2014). Here, the diets affected the *srebp2* gene expression with a higher expression in the group fed less fish oil (significant in 10FO/HIGH *vs*. 75FO/HIGH), indicating higher biosynthesis of cholesterol in the low‐fish‐oil diets.

The last step in the synthesis of DHA involves a chain shortening of 24:6n‐3 to DHA by the enzyme acyl‐CoaA oxidase. There were no significant differences in the gene expression of *acox* (Table 6) that could have explained the differences in hepatic DHA content of the four groups. There were also no effects of either diets or family background on *cpt1* gene expression level involved in mitochondrial β‐oxidation (Table 6). In conclusion, the differences in diet and family background resulted in different hepatic fatty acid compositions and differences in the expression of several lipid key genes. These findings are largely in agreement with previous studies.

### 4.2 Putative regulatory roles of DE miRNAs in *S. salar* lipid metabolism

The analysis of relative expression differences revealed 12 mature miRNAs with changed expressions. Some of these miRNAs were differentially expressed when comparing family groups, others were differentially expressed when comparing differences in diets and the expression of four miRNAs was associated with both family groups and diet. As the expression of several miRNAs was affected by family background independent of diet, the initial selection of the parental individuals in two family groups using *Δ6fad_b* expression levels led to a selection into two family groups that also differed in their expression of certain miRNAs. Nonetheless, as the diet affected the expression of six miRNAs, their expression seems to be modulated by dietary levels of fish oil and vegetable oil.

One species‐specific mature miRNA (ssa‐miR‐nov1‐5p) and the teleost‐specific miR‐462b‐5p were among the DE miRNAs, indicating they may have regulatory roles in the salmon lipid metabolism. All the remaining 10 evolutionary‐conserved DE miRNAs have been identified as miRNAs that are important in lipid metabolism and/or adipogenesis in other vertebrates (Ahn *et al*., 2013; Arner & Kulyte, 2015; Chen *et al*., 2014; Cheng *et al*., 2018; Hu *et al*., 2012; Shin *et al*., 2014; Ye *et al*., 2014). In most cases their particular functions have not been experimentally established but rather assumed from target gene predictions. Nonetheless, the finding that same evolutionary‐conserved orthologous miRNAs are discovered as differentially expressed in other vertebrate studies of lipid metabolism indicates that they may have similar regulatory functions in Atlantic salmon. The miRNA ssa‐miR‐135c‐5p (identical to 135a‐5p in Chen *et al*.) controlled, *e.g*., adipogenesis and lipid droplet accumulation in a mammalian cell line study (Chen *et al*., 2014). Also, ssa‐miR‐146a (identical to miR‐146b in Ahn *et al*., 2013) was reported as a regulator of adipogenesis by suppressing the SIRT1‐FOXO cascade.

*In silico* predictions of target genes are a common approach to further elucidate the role of a certain DE miRNA. One limitation of such predictions when studying non‐model species is that the 3′UTRs of most genes, including those genes suggested as targets in other vertebrate studies, are poorly characterized. Also, a large proportion of the predicted targets will be false positives (Andreassen & Hoyheim, 2017). Nevertheless, such predictions may narrow down the genes relevant to study further by experimental approaches. One finding in the present *in silico* analysis was that ssa‐miR‐137a‐3p was predicted to target *cdc42*. If there is a conserved miRNA/target interaction in vertebrates, one would expect that the target site sequence in the 3′UTR of the target transcript is also conserved across species. Interestingly, the same miRNA ortholog (miR‐137) has been suggested by Shin *et al*. to control adipogenesis in human adipose cell lines, also by targeting the same orthologous target transcript (*cdc42*) (Shin *et al*., 2014).

When there is a lack of evidence from comparative studies of particular miRNA–target interactions, direct measurements of the expression changes in the predicted targets could add evidence that they are true targets. One predicted DE miRNA–target gene pair did reveal reciprocal change in abundance in one group comparison (ssa‐miR‐203a‐3p and *srebp‐*1), whereas the others did not. Nonetheless, the negative regulation mechanism executed by the miRISC when directed to a target gene by the guide miRNA depends on the kind of Argonaute homologue that are part of RISC. There are several Argonaute proteins in fish, and whereas one slices the target transcript (leading to degradation of the target mRNA), the others lead to repression of target gene protein expression by, *e.g*., translational inhibition. The Argonaute proteins are not well characterized in Atlantic salmon, but investigations in other teleosts indicate that cleavage of the target mRNA is not the dominant regulatory mechanism in fish (Chen *et al*., 2017). Thus, although one predicted DE miRNA–target gene interaction showed signs of reciprocal change in abundance in the groups compared as expected for RISC‐mediated degradation of target mRNA, the absence of such relationship in the other predicted DE miRNA–target gene pairs does not rule out that they are true targets. This is also the case for the two genes *acox1* and *cp1* as the mRNA expression, not protein expression, was investigated here.

Some orthologues of the DE miRNAs identified are known to be important, not only in lipid metabolism and adipogenesis but also in low chronic inflammatory processes associated with pathological accumulation of lipids in vertebrates (Arner & Kulyte, 2015). The 10FO diet group, and particularly the 10FO/LOW group, showed a significantly lower percentage of the anti‐inflammatory EPA (Table 3). Reduced omega‐3 levels and increased proinflammatory omega‐6 fatty acid levels may be associated with metabolic imbalance in the liver (Scorletti & Byrne, 2013). Interestingly, the miRNAs ssa‐miR‐125a‐3p and ssa‐miR‐221 and the three miR‐146 family members are all associated with inflammation in the adipose tissue (Arner & Kulyte, 2015). For example, Mir‐146a is shown to inhibit oxidized low‐density lipoprotein‐induced lipid accumulation and inflammatory response by targeting toll‐like receptor 4c (Chen *et al*., 2016; Yang *et al*., 2011). All these miRNAs, as well as the teleost‐specific ssa‐miR‐462b‐5p, showed changed expressions in the inflammatory phase of viral disease in Atlantic salmon (Woldemariam *et al*., 2020). Whether the observed differential expression in these particular miRNAs and in the relative level of EPA could influence the immune response capacity of the groups would be an interesting topic for future work.

In summary, applying the 12 DE miRNAs as an input in target gene predictions, a limited number of key genes in the Atlantic salmon hepatic lipid metabolism were predicted as putative targets. One of these miRNA–target gene interactions was supported by a similar study in mammals. More experimental studies in Atlantic salmon are, nonetheless, needed to validate and fully understand the predicted miRNA–target gene interactions. Such knowledge may further help understand whether the identified DE miRNAs participate in the regulatory networks that control lipid metabolism in Atlantic salmon.

## AUTHOR CONTRIBUTION

Conceived and coordinated the study: R.A. and T.‐K.K.Ø.; methodology: R.A. and T.‐K.K.Ø.; software: R.A. and N.T.W.; validation: R.A. and N.T.W.; formal analysis: R.A., T.‐K.K.Ø., C.E.L., G.M.B. and N.T.W.; investigation: R.A. and N.T.W.; resources: R.A. and T.‐K.K.Ø.; data curation: R.A., N.T.W. and T.‐K.K.Ø.; writing – original draft preparation: T.‐K.K.Ø., N.T.W. and R.A.; writing – review and editing: R.A., T.‐K.K.Ø., B.R., G.M.B. and N.T.W.; visualization: R.A., N.T.W. and T.‐K.K.Ø.; supervision: R.A. and T.‐K.K.Ø.; project administration: R.A.; funding acquisition: R.A. All authors revised and approved the final draft.

## Supporting information


**Supplementary File S1** Results from RNA extraction

**Supplementary File S2** Forward primer sequences for RT‐qPCR of miRNAs, efficiency and melting curves

**Supplementary File S3** Primers for qPCR of predicted target genes, efficiency and melting curves

**Supplementary File S4** Descriptive data from the small RNA sequencing and FASTQC results

**Supplementary File S5** DE miRNAs revealed by DESeq2 analysis

**Supplementary File S6** *In silico*‐predicted target genes
