# Supplementary material for: Modulation of hepatic miRNA expression in Atlantic salmon (Salmo salar) by family background and dietary fatty acid composition
Source: J Fish Biol. 2021 Jan 11;98(4):1172–85. doi: 10.1111/jfb.14649 (PMC8048513; doi:10.1111/jfb.14649)
Supplement: Supplementary file 4 — Supplementary File S4 Descriptive data from the small RNA sequencing and FASTQC results [file JFB-98-1172-s006.pdf]

Table 4. Descriptive data from small RNA sequenced samples

Group ID[a]	sample ID[b]	Conc. (ng/µl)	A260/280	A260/230	Total # reads[c]	Trimmed, filtered reads[d]	Reads mapped as miRNAs[e]
10FO/HIGH	37_260_201_10+	1034	2,16	2,25	10867580	7260044 (66,8)	6541369 (84,3)
10FO/HIGH	38_268_200_10+	1142	2,16	1,99	13040194	10063400 (77,1)	9271037 (87,5)
10FO/HIGH	39_288_203_10+	978	2,16	2,22	12384277	8222141 (66,4)	7457703 (84,7)
75FO/HIGH	40_247_200_75+	795	2,13	1,91	13978045	9617076 (68,8)	8703420 (83,9)
75FO/HIGH	41_249_201_75+	1037	2,13	2,08	12001218	7830378 (65,2)	7097692 (83,1)
75FO/HIGH	42_254_203_75+	1689	2,15	2,15	10484738	7773933 (74,1)	7110075 (85,9)
10FO/LOW	43_264_205_10-	877	2,15	2,13	13168626	8688864 (65,9)	7906073 (84,8)
10FO/LOW	44_266_206_10-	1012	2,16	2,21	12034086	8797824 (73,1)	7955590 (84,8)
10FO/LOW	45_291_207_10-	581	2,14	2,21	6650167	421844 (66,5)	4012084 (85,8)
75FO/LOW	46_252_206_75-	1341	2,16	2,16	12559127	9155506 (72,9)	8228745 (84,0)
75FO/LOW	47_277_205_75-	1085	2,16	2,19	11239435	8668656 (77,1)	7923407 (86,0)
75FO/LOW	48_257_206_75-	1216	2,17	2,11	10601873	8034237 (75,8)	7263761 (85,0)

[a]Groups fed either 10FO or 75FO diet and from genetic groups selected for high (HIGH) or low (LOW) expression of the desaturase isomer *Δ6fad_b*.

[b]Sample ID corresponds to the size filtered and adapter processed FASTQC file below

[c]Total number of reads from the Illumina smallRNA sequencing of the sample

[d]Total number of reads after removal of adapter and size filtering (reads <18 nt's and > 24 nt's were removed)

[e]Number of reads that were mapped as miRNAs
